# Supplementary material for: A novel approach to decision making in rice quality management using interval-valued Pythagorean fuzzy Schweizer and Sklar power aggregation operators
Source: PLoS One. 2024 Oct 24;19(10):e0311525. doi: 10.1371/journal.pone.0311525 (PMC11500917; doi:10.1371/journal.pone.0311525)
Supplement: S1 Dataset — (PDF) [file pone.0311525.s001.pdf]

Manuscript ID: PONE-D-24-07498R1 - [EMID:81dd6c40939b0989]
Title: A Novel Approach to Decision Making in Rice Quality Management using Interval-Valued Pythagorean fuzzy Schweizer and Sklar Power Aggregation Operators

---

1. Introducing a Interval-Valued Pythagorean fuzzy, defined aggregation operators, set operations, and algorithm for decision making and elaborating versatility of developed aggregation operator structure through applications.

Model: A methodology for solving MADM problems using Interval-Valued Pythagorean fuzzy Schweizer and Sklar Power Fuzzy (IVPF) SS operators is presented in this manuscript.

Methods/Aggregation Operators: By utilizing the SS norms and adding a parameter $\eta < 0$, the interval-valued Pythagorean fuzzy Schweizer and Sklar power aggregation operators improve existing aggregation operators. With a more exact and customized arrangement of distinct assessments, this parameter gives the aggregation procedure more fine-tuning capability. When dealing with complicated and varied decision-making difficulties, interval values are especially helpful since they guarantee improved accuracy and handling of uncertainty. The real essence of the actual information can be reflected in more informative and dependable outputs by using these operators.

Software: we use four different software.

- Overleaf: for writing (https://www.overleaf.com/project)
- Mathematica & Powerpoint: for plotting graphs (https://www.wolfram.com/mathematica/online/)

---

2) The following descriptions serve as explanations for the figures illustrating to decision making in rice quality management

Graph values:

In "Figure 1", we express graphical model for solving MADM problem.

In "Figure 2", we express the parametric sensitivity by altering the values of $\eta < 0$ using IVPFSSPWA operator through the score values of Table 3.

In "Figure 3", we express the parametric sensitivity by altering the values of $\eta < 0$ using IVPFSSPWG operator through the score values of Table 4.

In "Figure 4", we express the parametric sensitivity by different known weight vectors using IVPFSSPWA operator through the values of Table 5.

In "Figure 5", we express the parametric sensitivity by different known weight vectors using IVPFSSPWG operator through the values of Table 6.
