# Supplementary material for: A novel approach to decision making in rice quality management using interval-valued Pythagorean fuzzy Schweizer and Sklar power aggregation operators
Source: PLoS One. 2024 Oct 24;19(10):e0311525. doi: 10.1371/journal.pone.0311525 (PMC11500917; doi:10.1371/journal.pone.0311525)
Supplement: S1 File — (PDF) [file pone.0311525.s002.pdf]

Data Availability statement

All the relevant data are within the manuscript.

Corresponding Author: Muhammad Ahsan Binyamin
Department of Mathematics, Government College University Faisalabad 38000, Pakistan
Email: mahsanbinyamin@gcuf.edu.pk
