# Supplementary material for: A novel approach to decision making in rice quality management using interval-valued Pythagorean fuzzy Schweizer and Sklar power aggregation operators
Source: PLoS One. 2024 Oct 24;19(10):e0311525. doi: 10.1371/journal.pone.0311525 (PMC11500917; doi:10.1371/journal.pone.0311525)
Supplement: Attachment — Submitted filename: Response to Reviewer Comments File.pdf [file pone.0311525.s004.pdf]

Original Manuscript ID: PONE-D-24-07498

Original Article Title: "A Novel Approach to Decision Making in Rice Quality Management using Interval-Valued Pythagorean fuzzy Schweizer and Sklar Power Aggregation Operators"

To: Editor PLOS ONE

Re: Response to reviewers

Dear Editor,

Thank you for allowing a resubmission of our manuscript, with an opportunity to address the reviewers' comments.

We are uploading (a) our point-by-point response to the comments (below) (response to reviewers), (b) an updated manuscript with highlighting indicating changes, and (c) a clean updated manuscript without highlights (PDF main document).

Best regards,

M. Ahsan Binyamin

Reviewer#1, Concern # 1: The abstract must be more precise and focus on the objective: Why is the study needed?

Author response: Thank you for your valuable suggestion.

Author action: We have described all those points highlighted by you along with focusing on main objective behind this study.

Reviewer#1, Concern # 2: Brief results must be shown in the Abstract, and at present, it is missing.

Author response: Thank you so much for your valuable suggestion.

Author action: We have shown the main results in the Abstract.

Reviewer#1, Concern # 3: Literature review - Please improve on this, and state clearly what the focus of the paper is and why the focus is presented. What importance it brings to the study line is not clear.

Author response: Thank you for your suggestion.

Author action: We have improved our literature review and also described motivations about my environment and proposed oprerators.

Reviewer#1, Concern # 4: There is a clear lack of motivation as to why the authors chose these aggregation operators in an interval-valued Pythagorean fuzzy environment.

Author response: Thank you for your valuable suggestion.

Author action: We have already elaborated this in the motivation subsection.

Reviewer#1, Concern # 5: Please bring some facts and figures in the introduction to support the ideas about neutrosophic sets and other fuzzy sets such as: (a): Comparison between fuzzy soft matrix (fsm) and interval valued fuzzy soft matrix (ivfsm) in decision making. (b): Aggregation Operators for Interval-Valued Pythagorean Fuzzy Soft Set with Their Application to Solve Multi-Attribute Group Decision Making Problem. (c): Einstein aggregation operators for Pythagorean fuzzy soft sets with their application in multiattribute group decision-making. (d): An integrated group decision-making technique under interval-valued probabilistic linguistic T-spherical fuzzy information and its application to the selection of cloud storage. (e): An interaction and feedback mechanism-based group decision-making for emergency medical supplies supplier selection using T-spherical fuzzy information. (f): An Intelligent MCGDM Model in Green Suppliers Selection Using Interactional Aggregation Operators for Interval-Valued Pythagorean Fuzzy Soft Sets. (g): Extension of correlation coefficient based TOPSIS technique for interval-valued

Pythagorean fuzzy soft set: A case study in extract, transform, and load techniques. (h): A Decision-Making Approach Based on Score Matrix for Pythagorean Fuzzy Soft Set.

Author response: Thank you for the recommendation.

Author action: We have done it by adding all the above citations.

Reviewer#1, Concern # 6: There are many symbols, so please explain all of them clearly to help readers understand.

Author response: Thank you for suggestion.

Author action: We have tried to reduce the abbreviations in this manuscript.

Reviewer#1, Concern # 7: Missing the definitions of fuzzy sets and Pythagorean fuzzy sets in the preliminaries section.

Author response: Thank you for your valuable suggestion.

Author action: We have added them in the preliminaries section.

Reviewer#1, Concern # 8: What is the source of the data used in section 5?

Author response: Thank you for asking.

Author action: We have used rough data instead of real data in section 5.

Reviewer#1, Concern # 9: Provide a better validation section with comparisons with the existing models. Add more discussion on the results and discuss advantages and limitations.

Author response: Thank you for your valuable suggestion and careful review of the article.

Author action: We have applied validation test in the comparison section that are shown the advantages of our operators and also discussed limitations.

Reviewer#1, Concern # 10: Comparison can be made effective, too, with more results and discussion and also expand the experimental setup.

Author response: Thank you for your valuable suggestion and careful review of the article.

Author action: We have used validation to create our comparison effectively.

Reviewer#1, Concern # 11: The authors need to explain the contribution and objectives of this research in a wide manner.

Author response: Thank you for your valuable suggestion.

Author action: We have tried our best to explain them (see in author's list).

Reviewer#1, Concern # 12: Findings, limitations, and recommendations of this paper can be discussed more in the conclusion section.

Author response: Thank you for your valuable suggestion.

Author action: We have mentioned limitations above the conclusion section and have tried to discuss more in the conclusion section.

Reviewer#1, Concern # 13: Please bring and focus on future research directions.

Author response: Thank you for your valuable suggestion.

Author action: We have added the future directions clearly.

Reviewer#1, Concern # 14: Some sentences in the text need to be rewritten. The manuscript needs to be carefully edited by professional English editors, paying special attention to English grammar, spelling, and sentence structure so that readers can clearly understand the objectives and results of the research.

Author response: Thank you for your valuable suggestion.

Author action: We have tried to rectified them.

Reviewer#2, Concern # 1: The introductory section must appropriately highlight the motivations and objectives of this research by reporting what are the performance improvements compared to well-known fuzzy multi-criteria decision-making in the literature..

Author response: Thank you for your valuable suggestion.

Author action: We have mentioned motivation in the introduction section and also have improved literature.

Reviewer#2, Concern # 2: Clearly articulate why "interval-valued Pythagorean fuzzy " was chosen as the research topic and highlight its practical relevance. What are the advantages?

Author response: Thank you for the suggestion.

Author action: Because this environment is an extension of Pythagorean fuzzy set and interval valued fuzzy set and have described its importance in the motivation subsection.

Reviewer#2, Concern # 3: There are too many redundant references in the introduction, some of which can be deleted. There are citations that comprise of multiple references for eg. ([6];[7];[8];[9]), ([10];[11];[12];[13]), ([23];[24];[25]). It is advised to refer to each reference individually in the text rather than grouping them together.

Author response: Thank you for the suggestion.

Author action: We have successfully removed all bulk references in the introduction section.

Reviewer#2, Concern # 4: The main contribution of the manuscript should be listed in bullets under the Introduction section.

Author response: Thank you so much for your valuable suggestions.

Author action: We have done it accordingly.

Reviewer#3, Concern # 5 Several aggregation operators exist in the literature, such as Dombi, Archimedean, Frank etc. why authors select the Schweizer and Sklar power aggregation operators is not clear. Please refer to and cite properly to demonstrate the superiority of your work. q-Rung Orthopair Fuzzy Archimedean Aggregation Operators: Application in the Site Selection for Software Operating Units; Intuitionistic fuzzy Dombi aggregation operators and their application to multiple attribute decision-making; Q-rung orthopair fuzzy Frank aggregation operators and its application in multiple attribute decision-making with unknown attribute weights. What are the advantages?

Author response: Thank you so much for your suggestion.

Author action: We have mentioned the advantages of our operators using SS norms in the motivation section and also have mentioned all above citations.

Reviewer#2,Concern#6: In Step 6 and 7 of Algorithm of Section 4 if score values become equal what will happen? Modify the algorithm with the use of accuracy function.

Author response: Thank you for suggestion.

Author action: When the score values are same then we use the accuracy function for ranking system. We have modified our algorithm according to it.

Reviewer#2,Concern#7: Remove background fill of Fig. 1..

Author response: Thank you for suggestion.

Author action: We have removed it.

Reviewer#2,Concern#8: In section 5 Step 1 is written as "Data collection in the matrix form (For q=3) in table [1]". Why q=3?

Author response: Thank you for highlighting.

Author action: This was the typo error and we have made it correct by removing it.

Reviewer#2,Concern#9: In Tables 5 & 6 of Sensitivity analysis how new sets of weights are generated? Mention.

Author response: Thank you for asking.

Author action: Basically, here we have used known weight vectors whose sum is exactly equal to one and have also mentioned in our algorithm.

Reviewer#2,Concern#10: Comparative analysis needs to be stronger. I hope the author can add more comparisons to prove the rationality and effectiveness of the proposed method. Table 7 shows that the ranking obtained by the proposed method and the comparative method are identical. Therefore, it is imperative to ponder why decision-makers should choose this method over existing ones.

Author response: Thank you for suggestion.

Author action: We have performed validation test to improve our comparison section.

Reviewer#2,Concern#11: Your method could be applied to another extension of fuzzy sets such as 'quasirung fuzzy sets‟. Include these in the future scope of the conclusion section by properly citing the following

references. https://doi.org/10.1016/j.engappai.2022.105299; https://doi.org/10.1007/s41066-021-00308-9

Author response: Thank you for suggestion.

Author action: We have mentioned this environment with these citations.

Reviewer#2,Concern#12: English writing should be improved considerably throughout the manuscript.

Author response: Thank you for suggestion.

Author action: We have tried to improve it.
